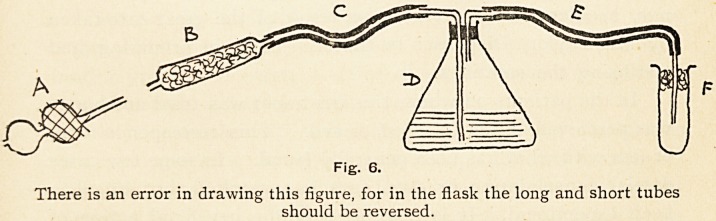# Aneurysm of the Ascending Aorta

**Published:** 1904-03

**Authors:** F. H. Edgeworth

**Affiliations:** Assistant Physician to the Bristol Royal Infirmary


### ANEURYSM OF THE ASCENDING AORTA.

BY

F. H. EDGEWORTH, M.B., D.Sc. (Lond.),

*Assistant Physician to the Bristol Royal Infirmary.*

CASES of aneurysm of the ascending aorta are generally divided into two groups—those affecting its very root, and those which arise from its next portion, that between the sinuses of Valsalva and the origin of the innominate artery.

The three cases recorded in this paper belong to the second class. Their exceptional character consisted in the fact that they arose from the concave side of the ascending aorta and gave evidence of their presence on the left side of the sternum, whereas the great majority arise from the convex side of the aorta and grow to the right. Cases of this character are so rare, that they are not even mentioned in some treatises on diseases of the heart and aorta, *e.g.* in those by Bramwell and Gibson, and they present some difficulties in diagnosis which are not experienced in the more usual type of case.

Mrs. A., aged 40, a patient of Dr. Martin's, was admitted to the Infirmary on August 10th, 1901, complaining that for three weeks she had had a sensation of a "throbbing lump" in the chest, which was worse after exertion and better after a night's rest. She had had rheumatic fever ten years previously, but no other illness, and there was no history or evidence of syphilis or malaria. She had had six healthy children, and one born dead at full term (a breech presentation). Her occupation was that of a housewife, and was not laborious.

On examination of the chest, the apex-beat could be felt just inside the nipple in the fifth intercostal space; there was no general ventricular impulse and no thrill. On percussion, the superficial cardiac dulness was found to be normal. In the second left interspace, extending from the left border of the sternum to one inch outside it, was an area which was dull on percussion and pulsated with an impulse slightly later in time than the apex-beat (Fig. 1). This barely-visible impulse was easily palpable; it was smooth, without any vibratile character, and as it died away from under the finger no diastolic shock could be felt. The area of dulness and pulsation did not become appreciably less when the patient made a deep inspiration. There was no dulness on percussion over the sternum or in the third left intercostal space. The rate of the heart-beats was 84 per minute, and they were regular in time and force. The radial pulses were synchronous, of the same rate as the heart-beats, and easily compressed; the brachial and radial arteries were not thickened. There was no tracheal tugging. On auscultation the cardiac sounds were normal at the apex; at the right base the second sound was slightly accentuated. Over the dull area in the second left intercostal space a rough, low-pitched murmur could

Fig. 1.

be heard synchronous with the palpable impulse, followed by the accentuated second sound. The lungs and abdomen were normal. The urine contained a trace of albumin. There was no œdema of the legs or other part of the body, and the patient was not anæmic. The larynx was normal. On skiagraphic examination by Mr. Taylor, a pulsating bulge to the left of the ascending aorta could be seen, corresponding in position to the area mentioned above, though extending a little further outwards.

The patient was put to bed and kept in the horizontal position. Solid food was not restricted, but she was asked to take as little fluid as possible. Twenty grains of iodide of potassium were given three times a day, from August 10th to November 5th.

During the month of September the aneursym increased slowly in size. Thus, on November 1st, the area of dulness and pulsation in the second left intercostal space extended to the distance of two inches from the left border of the sternum, and had spread into the first space, whilst the transverse aorta had been pushed a little upwards, and could be felt beating in the episternal notch, and the apex-beat was now one inch outside the nipple in the fifth space (Fig. 2).

Fig 2

Injections of gelatine were begun on November 5th, and were given twice a week from that time until the patient left the Infirmary on January 10th. By that time twenty injections had been made; there was, however, absolutely no result, the physical signs being precisely the same as they had been on November 1st.

I have heard that the patient remained in bed for about a year after, taking iodide continuously, and then gradually got about. The physical signs remain the same.

W. B., aged, 37, was first seen on January 13th, 1898. He had had gonorrhœa, but not syphilis, nor any other illness. For some months he had been troubled by a cough. On examination of the chest, it was found that the apex-beat of the heart was in the fifth space, half an inch inside the nipple line. The rate was 66 per minute, and regular. In the second left intercostal space, extending outwards about an inch from the left border of the sternum, pulsation slightly later in time than the apex-beat was visible and palpable. On percussion, this area was dull, whilst the third intercostal space was resonant on light percussion (Fig. 3). The radial pulses were synchronous, regular, of the same rate as the cardiac-beats, and of moderate tension. The brachial and radial arteries were not atheromatous. On auscultation, the cardiac sounds were normal at the apex, the second sound on the right of the sternum was not accentuated, whilst on the dull area and for half an inch round it a loudish, rough, systolic murmur was audible, followed by a second sound barely

Fig. 3.

Fig. 4.

different from that heard to the right of the sternum. There was no evidence of pressure on any intrathoracic structure; in particular, the vocal cords were normal. The lungs, abdomen and urine were normal.

Iodide was prescribed and absolute rest advised.

Six months later the aneurysm had increased in size, extending into the first and third left intercostal spaces (Fig. 4). Further, the systolic pulsation was attended by a thrill, the systolic murmur could be heard over the back as well as over the pulsating area, and the second sound was lower in pitch over the aneurysm than on the right side of the sternum. There was still no sign of pressure on veins, nerves, or air-passages within the thorax.

The patient passed from under my observation, but eventually, I believe, got quite well.

No skiagraphic examination was made.

E. C., aged 37, a sergeant in the army, was admitted to the Infirmary on August 28th, 1902, at the request of Dr. Wallace. He had had for some five months a sense of discomfort in the chest, especially when lying on the left side. Sixteen years previously he had had a venereal sore, which, as far as he could remember, was not followed by any "secondary" symptoms. Otherwise he had been healthy. For three months he had been kept in bed on a restricted fluid diet, and had been given large doses of iodide without any improvement resulting.

Fig. 5.

The condition on admission was as follows. The right side of the chest was normal. On the left side the apex-beat was in the fifth space, one inch outside the nipple, regular in rate, 80 per minute. In the first, second and third left intercostal spaces was an area of dulness on percussion, reaching from the left border of the sternum outwards for the extent, in the third space, of four inches. This dull area was continuous with that of the heart below (Fig. 5). This dull area visibly pulsated, and on palpation each pulsation, slightly later in time than the apex-beat, was felt to be vibratory in character, and the systolic thrill was immediately followed by a diastolic shock. The radial pulses were equal and synchronous, and easily compressible. There was distinct atheroma of the brachial and temporal arteries. No tracheal tugging was present, nor any evidence of obstruction of veins, arteries, trachea, bronchi, œsophagus, or sympathetic nerves within the chest. The vocal cords were normal.

On auscultation, a rough systolic murmur could be heard all over the front of the chest, obscuring the first cardiac sound, though loudest over the dull, pulsatory area above the heart. It could also be heard over the back of the chest on the left side. An accentuated second sound could be heard on the right of the sternum, and also, but of lower pitch, on the left side over the pulsating area. The lungs, abdomen, and legs were normal. The urine had a sp. gr. of 1020, and contained a small quantity of albumin.

The treatment consisted of rest in bed, restriction of fluids, and the administration of 20 grains of iodide three times a day. In addition, gelatine injections were given twice a week, commencing on September 2nd, and they were continued until the patient went out in January.

I cannot say that any improvement took place; though the thrill accompanying the systolic pulsation disappeared for a few days at the end of September, it soon returned, and other physical signs remained unchanged.

The patient, though never having any pain, became in course of time very restless and miserable, and during the last two months of his stay in the Infirmary morphia had to be injected twice daily. I subsequently heard that he died three months afterwards with œdema of the legs.

The diagnosis in the second case (on the second occasion that I saw him) and in the third case was simple and easy. In each there was a pulsating tumour on the left side of the upper part of the sternum, with a systolic thrill and murmur, diastolic shock and accentuated, low-pitched second sound over the tumour, some displacement of the heart, and no sign of involvement of the transverse portion of the aortic arch.

In the first case and in the first stage of the second case the diagnosis was somewhat of a difficulty, though confirmed in both either by skiagraphic examination or by subsequent events. There are two other conditions which may produce a pulsating patch of dulness accompanied by a systolic thrill in the innermost part of the second left intercostal space—anæmic dilatation of the heart and deficiency of the left border of the lung. The points of difference, however, are the following. In both conditions the dulness on percussion in the second left space continues downwards through the third space to join that of the superficial cardiac dulness in the fourth. In anæmic dilatation the systolic murmur is soft and blowing; there is in addition obvious pallor, and the patient is usually young. In the second condition the murmur is loud and rough, but usually disappears when the patient is told to take a deep breath and hold it; further, the individual is in perfect health and capable of prolonged exertion.

Examination with the X-rays and fluorescent screen in such doubtful cases is of great value and may serve to make a diagnosis certain, for only in aneurysm is a sacculated pulsating bulge of the aorta to be seen.

These cases of aneurysm, like those projecting to the right from the convex side of the ascending aorta, do not involve any other important thoracic organs, veins, arteries or nerves. They were instances of "aneurysm of physical signs" of Broadbent, in contradistinction to the "aneurysm of symptoms" occurring in the transverse portion of the aortic arch. In no one of the cases was any pain complained of. What attracted the patient's attention was a sensation of discomfort or throbbing.

The heart was not hypertrophied in the two first cases, the only change being slight cardiac displacement as the aneurysm slowly enlarged; this is what might have been expected, for there was no increase in the peripheral resistance. In the third case a moderate degree of atheroma was present, but as far as could be ascertained it had not caused hypertrophy of the heart, which was apparently merely a little displaced.

In all three cases the impulse over the aneurysm was distinctly later in time than the apex-beat, as could be easily ascertained by placing one forefinger on each spot. This is due to the fact that the apex-beat is not caused by the systole of the ventricle, but by the filling of the ventricle from the auricle.

Fig. 6.

There is an error in drawing this figure, for in the flask the long and short tubes should be reversed.

The method employed in preparing and administering the gelatine injections was as follows: 200 c.c. of distilled water are put into the flask D (Fig. 6), to this are added 1.4 grammes of common salt and 4.5 grammes of pure transparent white gelatine. On boiling the gelatine dissolves, forming a 2.25 per cent. solution. The flask is fitted with an indiarubber stopper, through which pass two small tubes, to each of which is attached a piece of thick-walled indiarubber aspirator tubing, C and E. To the other end of C is fixed a wide glass tube filled with sterilised cotton wool, whilst to E is attached the injection needle, which is packed round with sterilised cotton wool and inserted into the test-tube F. The apparatushas the merit that when it is heated the expanding air can escape at either end, and in subsequent cooling it is sucked back through the sterilised cotton wool. In this way, not only the gelatine solution but the air in the apparatus also can be made and kept aseptic.

The apparatus was put into an autoclave at a temperature of 120° C. for ten minutes on three successive days to sterilise the contents. When required, the gelatine flask was heated on a water bath to blood-heat; an indiarubber bellows A (such as is used in a freezing microtome) was then attached to B, the needle was taken out of the test-tube F and thrust deeply into the gluteal muscles of the patient, and the gelatine solution was slowly injected by compressing the bellows.

In the great majority of injections the patients had no pain beyond that caused by the prick of the needle; on one or two occasions some pain was experienced for an hour or two—possibly caused by the needle piercing a nerve. The usual result of an injection was a rise of temperature to 100° F. for a few hours, but there was never any inflammation or suppuration, nor, needless to say, tetanus—a result of the great care taken by Mr. Taylor, Dispenser to the Infirmary, in preparing and sterilising the solutions.

In the patients on whom this treatment was tried no change was perceived, either for good or evil. This corresponds more or less with what has been generally found. In some few cases partial consolidation or even cure has resulted, but this has been exceptional. It is possible that the beneficial action of gelatine is impaired or destroyed by the repeated sterilisation, which was carried out from remembrance of the fact that in some few cases tetanus has occurred.

And this liability is by no means small, for Tuck[1] has recently

[1] *J. Path. and Bacteriol.*, 1903, ix. 38.

discovered tetanus spores in eight out of fifteen samples of gelatine examined. He has also found that though tetanus spores are very resistant to the temperature of boiling water, yet one exposure of the gelatine solution to a temperature of 120° C. for ten minutes will absolutely kill them. It seems possible then that the failures above mentioned were due to unnecessarily repeated sterilisation of the solution.

### THE VAGARIES OF ABDOMINAL TUBERCULOSIS: A CLINICAL STUDY.

## Figures and Tables

**Fig. 1. f1:**
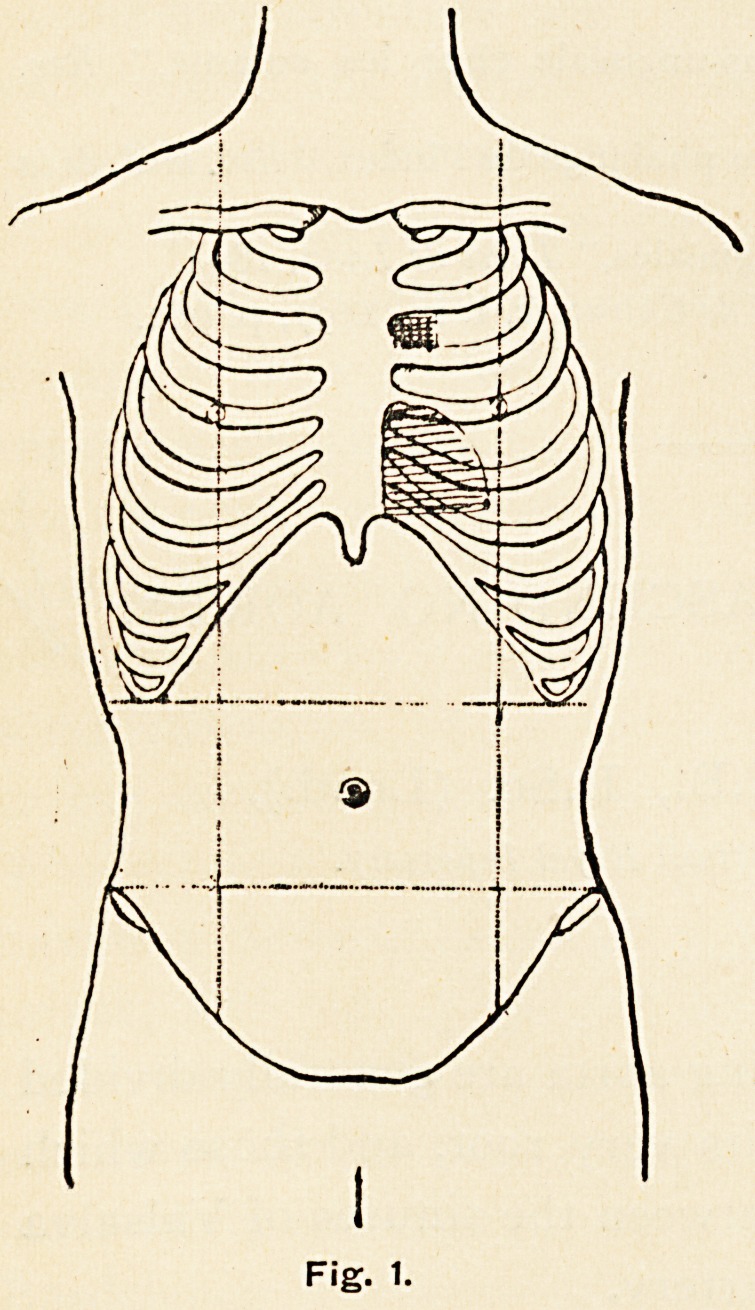


**Fig 2 f2:**
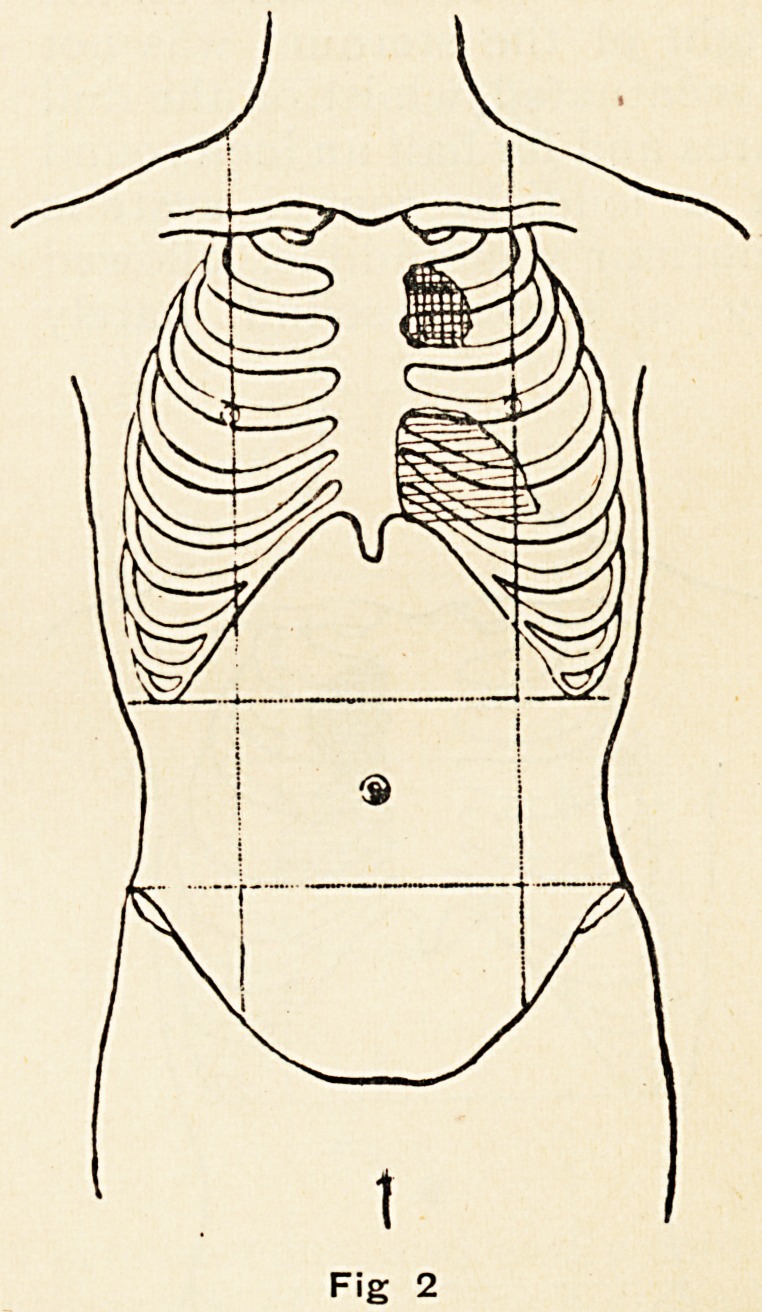


**Fig. 3. f3:**
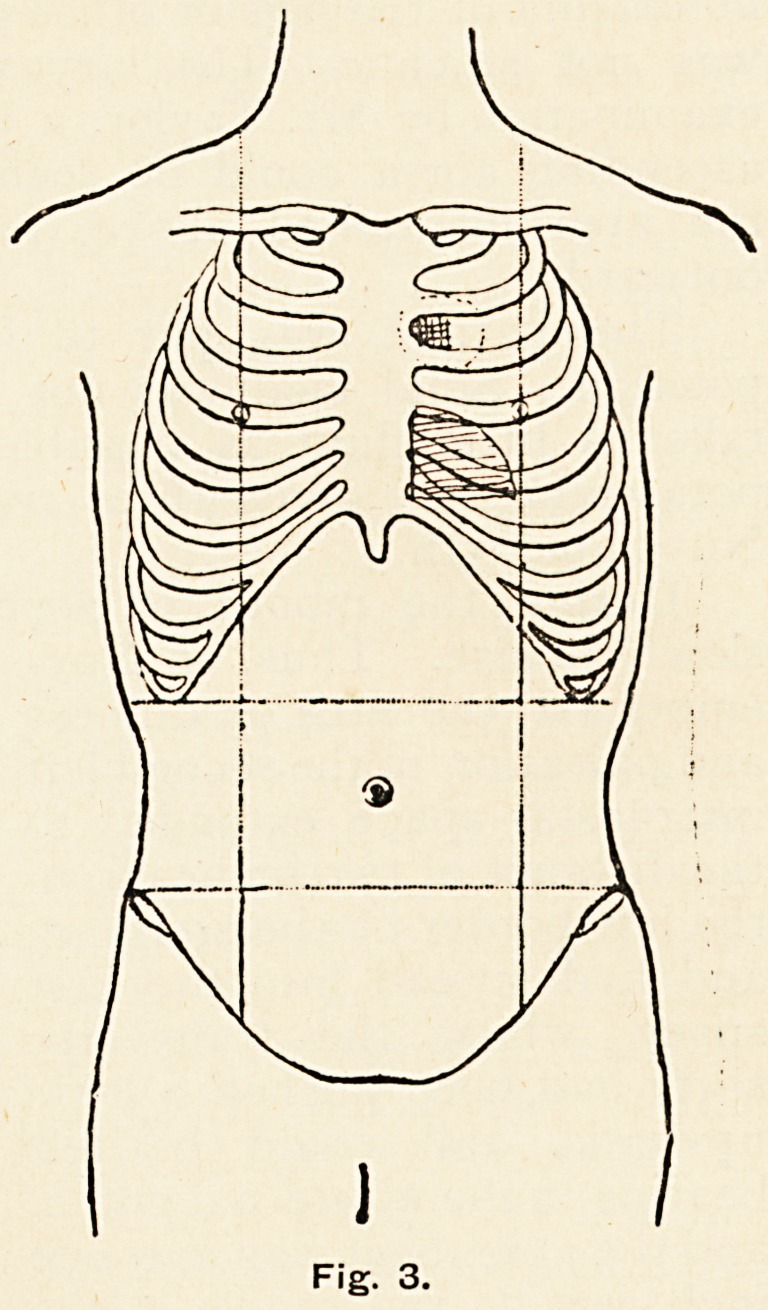


**Fig. 4. f4:**
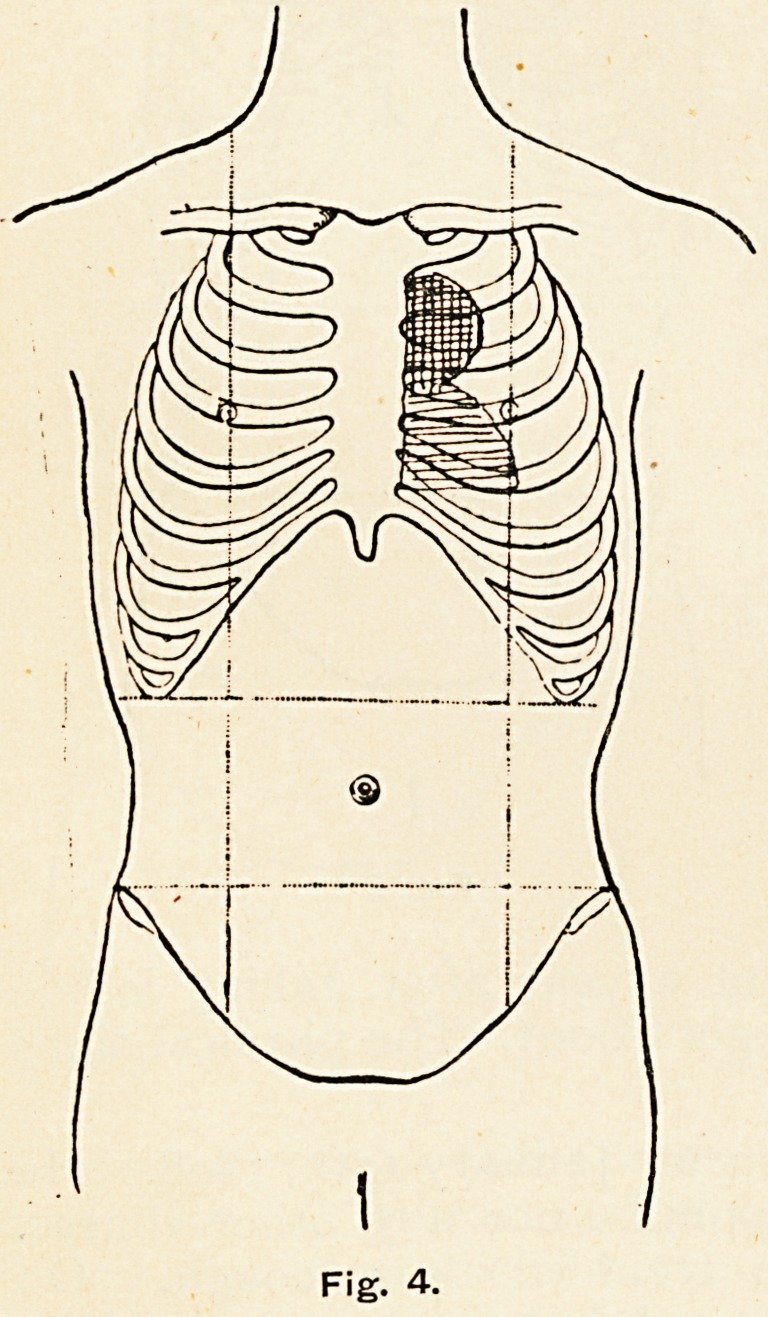


**Fig. 5. f5:**
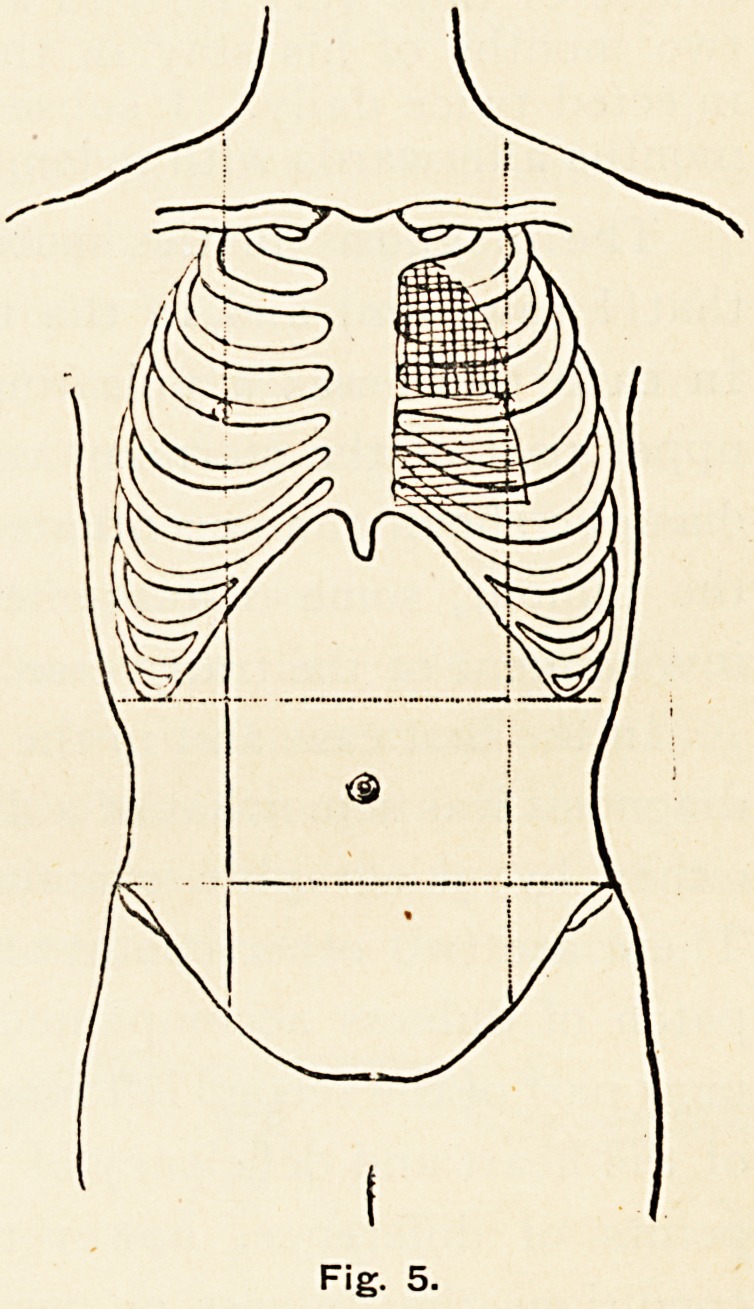


**Fig. 6. f6:**